# Transgenic tobacco plants constitutively expressing Arabidopsis *NPR1 *show enhanced resistance to root-knot nematode, *Meloidogyne incognita*

**DOI:** 10.1186/1756-0500-4-231

**Published:** 2011-07-04

**Authors:** D Bhanu Priya, N Somasekhar, JS Prasad, PB Kirti

**Affiliations:** 1 Department of Plant Sciences, University of Hyderabad, Hyderabad 500046, India; 2 Directorate of Rice Research, Rajendranagar, Hyderabad 500046, India

**Keywords:** Transgenic tobacco, *AtNPR1*, constitutive expression of PR proteins, nematode resistance, *Meloidogyne incognita*

## Abstract

In Arabidopsis, non-expressor of pathogenesis related genes-1, *NPR1 *has been shown to be a positive regulator of the salicylic acid controlled systemic acquired resistance pathway and modulates the cross talk between SA and JA signaling. Transgenic plants expressing *AtNPR1 *constitutively exhibited resistance against pathogens as well as herbivory. In the present study, tobacco transgenic plants expressing *AtNPR1 *were studied further for their response to infection by the sedentary endoparasitic root knot nematode, *Meloidogyne incognita*. Transgenic plants showed enhanced resistance against the root-knot nematode infection. Prominent differences in the shoot and root weights of wild type and transgenic plants were observed post-inoculation with *M. incognita*. This was associated with a decrease in the number of root galls and egg masses in transgenic plants compared to WT. The transgenic plants also showed constitutive and induced expression of some PR protein genes, when challenged with *M. incognita*.

## Discussion

Root-knot nematode species including *Meloidogyne incognita *are amongst the most destructive plant parasitic nematodes infecting almost all cultivated plants and are responsible for yield losses amounting to billions of dollars [1]. The disease is characterized by the presence of characteristic swellings called galls or root-knots on the roots of infected plants. Symptoms include stunted growth, wilting, and susceptibility to other pathogens leading to poor yields. The complex morphological and physiological changes that occur during the establishment of feeding sites by these intruders are reflected in altered gene expression in the host [2]. Molecular responses are similar in wounding as well as the stress caused by nematode infection and the consequent perturbations that are directed toward the initiation and maintenance of feeding sites [3].

Sedentary endoparasitic nematodes are obligate biotrophs that induce the formation of complex feeding sites within the roots of their plant host. This group of plant pathogens includes the root-knot nematodes (*Meloidogyne *spp.) and the cyst nematodes (*Heterodera *and *Globodera *spp.) that induce the formation of a group of fused cells called syncytium or giant cells in proximity to the root vasculature [4].

NPR1 (Non-expressor of pathogenesis related genes-1) has been characterized in great detail in the model system *Arabidopsis *and is shown to be a transcription activator with distinct protein-protein interaction motifs and possesses a bipartite nuclear localization signal (NLS). The *NPR1 *gene encodes a protein containing ankyrin repeat domain and a BTB/POZ (broad-*complex, tram track*, and *bricà-brac*/poxvirus, zinc finger) domain, both of which are involved in protein-protein interactions. The induction of systemic acquired resistance (SAR) triggers the monomerization of NPR1 and its activation. In the activated monomeric form, NPR1 crosses over to the nucleus and enhances PR gene expression [5] by interacting with the members of TGA family of b-ZIP transcription factors and stimulating their DNA binding activity. This allows the TGA factors to bind to cognate elements in promoters of certain genes and enhance their expression [5]. PR1 is a marker for the activated NPR1.

In several cases of nematode infection, plants elicit SAR pathway. *Heterodera schachtii *infection of *Arabidopsis *elicits salicylic acid independent upregulation of PR2 and PR5, and the PR1 expression was inversely correlated to nematode resistance. It was also concluded that SA acts via NPR1 to inhibit nematode parasitism which, in turn, is negatively regulated by SNI1 [6].

The transgenic plants expressing the *AtNPR1 *have been shown to exhibit enhanced resistance to both necrotrophic and biotrophic pathogens in various crops like tomato, rice, cotton, carrot etc. We have earlier reported the development and characterization of transgenic tobacco plants expressing *AtNPR1 *at molecular level and these plants showed enhanced tolerance to infestation by early instars of the generalist herbivore, *Spodoptera litura *[7] and oxidative stress tolerance induced by methyl viologen [8]. In the latter report, the transgenic lines were characterized again for the constitutive expression of *AtNPR1*, associated PR protein genes and some genes coding for antioxidant enzymes like ascorbate peroxidase and super-oxide dismutase. The transgenic lines did not show any differences in morphology, flowering and capsule formation indicating that the constitutive expression of *AtNPR1 *did not have any negative effect on the morphology or performance of plants (data not shown). Similar observations were made on the performance of wheat plants expressing *AtNPR1*, which showed that there was no reduction in the yield parameters like spike number and seed yield indicating that the *AtNPR1 *expression did not have any negative effect on the performance of wheat transgenic plants [9]. Lin et al. [10] have also reported that the transgenic tomato plants expressing *AtNPR1 *showed normal plant morphology and horticultural traits for at least four generations. Parkhi et al. [11] demonstrated that transgenic cotton plants expressing *AtNPR1 *exhibited enhanced tolerance to a semi-endoparasitic nematode, *Rotylenchulus reniformis *(reniform nematode) also along with resistance to infection caused by some fungal pathogens.

In this brief report, we show that the tobacco transgenic lines expressing *AtNPR1 *show enhanced resistance to the sedentary endoparasitic root knot nematode, *Meloidogyne incognita*.

We have used three different stabilized transgenic lines, 3-1, 17-1, 19-1 of tobacco expressing *AtNPR1*. Among these, 19-1 and 17-1 are high expression lines, where as 3-1 is a low expression line for the expression of the transgene, *AtNPR1 *[7]. An assay based on the number of root-galls and egg-masses produced by the nematode on host plant roots was used to evaluate the resistance of these transgenic plants for infection by the target nematode. Nematode culture for inoculation was obtained from pure cultures of the nematode maintained on tomato plants. About 40-day old-green house grown plants were inoculated with *Meloidogyne incognita*. Nematode inoculation was performed by introducing 250 second stage juveniles (J2) of *M. incognita *by making small holes in soil around the root system. These plants were maintained in the green house, and observations on plant growth parameters and nematode resistance were recorded six weeks after nematode challenge. In a repeat experiment, the transgenic line 19-1 has been tested for the number of root galls and egg masses upon challenge with the nematode and the results were similar to what have been documented here.

**Figure 1:**
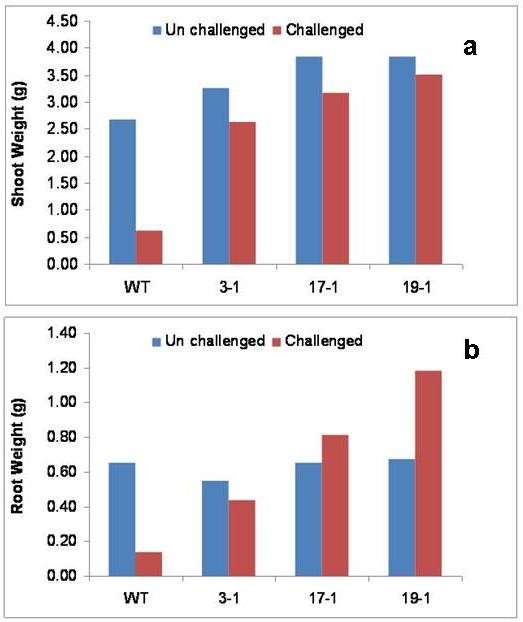
**Resistance to root-knot nematode *Meloidogyne incognita *in transgenic tobacco lines expressing *AtNPR1 *(a) Shoot weight and (b) Root weight after six weeks of inoculation with nematodes**. Transgenic plants show enhanced resistance to the nematode in all the parameters studied. The difference in means between the wild type and the transgenic lines was verified by Student's *t-*test and found to be significant at 5% level. Similar growth patterns in the unchallenged wild type and transformed plants were observed.

**Figure 2:**
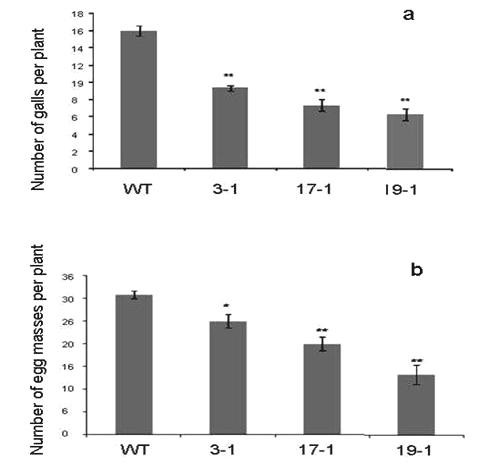
**Development of root knots (a) and egg masses (b) in the tobacco wild type and transgenic plants six weeks after infection with the nematode**. Note the significantly reduced root knots and egg masses in the transgenic plants expressing the *AtNPR1*. The graphs represent significance of mean differences between the WT and transgenic plants.

The results in present investigation documented superior performance of the transgenic tobacco lines expressing *AtNPR1 *compared to the wild type when challenged with the target nematode. When the growth pattern of different plants was analyzed six weeks after nematode inoculation, the transgenic plants expressing *AtNPR1 *showed better shoot and root growth when compared to wild type plants (Figures 1, 2). There was significant reduction in the vigor of the plants after infection with the nematode *M. incognita *in the case of the wild type plants. The *AtNPR1 *high expression line 19-1 recorded the highest shoot and root weight, which is about five folds higher, compared to the wild type plants. The number of root galls and egg-masses developed on the infected roots of the transgenic plants were significantly less (up to 50-60% less) compared to the wild type plants. Among the transgenic lines, lowest gall and egg-mass count was recorded in the high expression line 19-1 suggesting a dosage dependant effect of the expression of *AtNPR1*. The unchallenged wild type and transgenic plants exhibited similar root and shoot growth.

Observations on nematode development and plant growth parameters taken together suggest that the *AtNPR1*-expressing tobacco plants show better performance than the wild type when attacked by root-knot nematode, *M. incognita*. Further, the better performance of high expression transgenic lines over the wild type and low expression lines in terms of nematode resistance and plant growth observed in this study suggest that the expression levels of *AtNPR1 *can be positively correlated to the extent of resistance in the plants. Apparently, there were no phenotypic abnormalities and growth constraints in the transgenic plants expressing *AtNPR1*. This feature is in addition to the enhanced level of insect resistance already shown by the tobacco transgenic plants expressing *AtNPR1 *with respect to resistance to the early instars of the generalist herbivore, *Spodoptera litura *[7] and oxidative stress tolerance [8].

**Figure 3:**
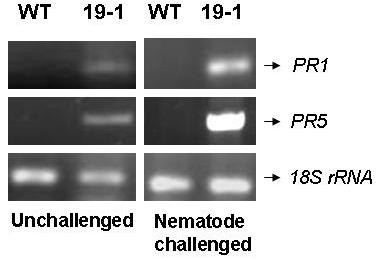
**Semi-quantitative RT-PCR based expression analysis of genes for PR protein genes in the wild type and transgenic plants**. PR1 and PR5 were studied in the high expression line 19-1. The transgenic line showed basal level of expression of PR1 and PR5, which got augmented after the treatment with the nematode.

Our results indicate a role for AtNPR1 in tobacco in limiting root knot nematode parasitism with a decrease in the formation of root galls and egg-masses. The high expression line, 19-1 showed high level of resistance to nematode inoculation when compared to other plants. This could be correlated with the constitutive expression of the pathogenesis related proteins and the genes for antioxidant enzymes like ascorbate peroxidase and super-oxide dismutase by the NPR1 transgenic plants [8]. Further, an analysis of the expression of genes for PR1 and PR5 in 19-1 transgenic plants by RT-PCR showed that they had basal constitutive level of expression, which was not observed in wild type plants. The gene expression got further enhanced upon treatment with the nematode (Figure 3). These observations indicate that the resistance to the nematode exhibited by the transgenic plants is associated with enhanced expression of genes for PR proteins.

Analysis of PR gene expression in wild-type plants inoculated with *H. schachtii *in *A. thaliana *revealed that PR-2 and PR-5, but not PR-1, were upregulated in roots following nematode infection [6]. However, PR-1 induction was observed in shoots of plants infected with *H. schachtii *suggesting that nematode infection elicits a SAR response in the plant, since PR-1 is commonly used as a molecular marker for SAR induction [3]. Tirumalaraju et al. [12] also showed the upregulation of genes for pathogenesis related proteins along with other genes in the comparative analysis of peanut cultivars resistant and susceptible to root-knot nematode (*Meloidogyne arenaria*). Montes et al. [13] analyzed the activities of peroxidase and superoxide dismutase in the roots of resistant and susceptible cultivars of wheat with respect to infestation by the nematode, *Heterodera avenae *and found that the resistant variety exhibited enhanced activities in comparison to the susceptible ones. Similarly, Simonetti et al. [14,15] also showed that ascorbate peroxidase and apoplastic peroxidase genes are upregulated in wheat lines resistant to *Heterodera avenae *compared to susceptible cultivars. Uehara et al. [16] made a comparative microarray analysis of the compatible and incompatible interaction in tomato cultivars with respect to the interaction with the potato cyst nematode (PCN; *Globodera rostochiensis*) at early stages. They observed that several genes like alocohol dehydrogenase, phenylalanine ammonia lyase and pyruvate decarboxylase were differentially regulated along with the pathogenesis related *PR1 *gene as a hallmark for the resistant cultivar against PCN. The nematode parasitism resulted in the inhibition of the SA signaling pathway in the susceptible cultivars.

Very recently, Hamamouch et al [17] showed that the infection by *Meloidogyne incognita *in *Arabidospsis *was associated with enhanced expression of *PR1, PR2 *and *PR5 *genes. It was also shown that infection with *M. incognita *activated both SA-and JA-dependent SAR in roots of Arabidopsis. Constitutive expression of PR-1 was shown to be associated with decreased susceptibility to infection by beet-cyst (*Heterodera schachtii*) and root-knot (*Meloidogyne incognita*) nematodes in Arabidopsis [17]. The constitutive basal expression as well as induced level of PR1 and PR5 in the present study along with the constitutive expression of genes for oxidative enzymes [8] was probably associated with enhanced resistance observed against the *M. incognita *in *AtNRP1 *expressing transgenic tobacco plants.

These observations demonstrate the versatility of NPR1 protein in providing multiple stress tolerance in crop plants. However, the actual mechanism by which AtNPR1 confers resistance to the target nematode remains to be worked out in detail.

## Material and methods

### Plant material

Transgenic tobacco plants expressing *AtNPR1 *were used in the investigation. They were analyzed in detail with regards to the transgene integration and expression, and at protein level [7]. They were also analyzed for oxidative stress tolerance and the constitutive expression of some genes has been documented along with the expression of *AtNPR1 *[8]. Stabilized lines of tobacco expressing *AtNPR*1, Viz., T3-1, T17-1 and T19-1 were used in the present investigation along with non-transformed plants, and they were treated with root knot nematode, *Meloidogyne incognita*.

### Seed germination and plant culture

All seeds are surface sterilized with sodium hypochlorite for 15 min and washed in sterile distilled water for 20 min. With the help of a cut pipette tip, the sterilized seeds were spread on petriplates containing half MS with 100 mg/ml kanamycin for transgenics and without kanamycin for non-transgenic control (wild type). The cultures were maintained at 28 ± 1°C under 16/8 h (light/dark) photoperiod with light supplied by cool white fluorescent lamps at an intensity of about 1600 lux for a period of 15 days for seed germination and sufficient seedling growth for transfer to sterile soil mixture.

All the plantlets raised *in vitro *were washed with sterile water and transferred to tea cups containing a sterile mixture of soil and vermiculite in a ratio of 1:1 for a period of 15 days in the green house. Subsequently, they were transferred to pots with sterile potting mixture of 1:1 ratio of soil and farmyard manure and maintained for 10 days.

### Nematode culture

Population of *Meloidogyne incognita *was maintained on susceptible tomato cultivar Pusa Ruby in a greenhouse. Nematode infected tomato plants were uprooted from culture pots and roots were washed free of soil under running water. Nematode egg masses were removed directly from tomato roots using forceps and placed on a modified Baermann funnel setup [18]. Second stage juveniles (J2) of *M. incognita *hatched in clean tap water were collected and used for challenge inoculation.

### Root-knot nematode resistance assay

An assay based on number of root-galls and egg-masses produced by the nematode on host plant roots was used to evaluate the resistance of transgenic plants. 40 day old-green house grown plants were inoculated with *Meloidogyne incognita*. Nematode inoculation was performed by introducing 250 second stage juveniles (J2) of *M. incognita *in soil around the root system of each plant. These plants were maintained in the green house, and observations on plant growth parameters and nematode resistance were recorded six weeks after nematode challenge. Six weeks after nematode inoculation, individual plants were uprooted, roots were washed free of soil, excess moisture removed with blotting paper, root and shoot portions separated, and fresh weight was recorded. Total number of galls and egg masses on entire root system of individual plants was counted by observing roots under Carl-Zeiss Stemi 2000C stereo-zoom microscope and the results are presented as number of galls or egg masses/plant. Three plants for each transgenic line along with the non-transformed wild type plants were inoculated with the juveniles of the target nematode and observations were made for the effect of the treatment on plant morphology. These experiments were repeated.

Simple Student's '*t*' test was used to analyze the significance of differences between the wild type and the transgenic plants.

### Semi-quantitative RT-PCR

Expression analysis of PR1 and PR5 genes was analyzed in the high expression line T19-1 using semi-quantitative RT-PCR following standard molecular biology protocols. Total RNA was isolated using the TRI-reagent (Sigma Aldrich, USA). MMLV reverse transcriptase (Sigma Aldrich, USA) was used for generating the first strand cDNA for RT-PCR. Primers used for the expression analysis of PR1 and PR5 genes are as follows:*PR1a *- Forward primer 5'CTTCTTGTCTCTACACTTCTC3' and reverse primer: 5'GCAAGA GACAACATATCCTC3', and *PR5*- Forward Primer 5'CTTGAGATCTTCTTTTG TTT TCTTC3' and reverse primer 5'ACTTCCAGGCATTTCCAAGGGAAA3' [19]. Amplification of 18S rRNA served as a control for equal loading.

## Competing interests

The authors declare that they have no competing interests.

## Authors' contributions

DB and NS have actually undertaken the investigations. JSP and PBK planned the investigation. We further state that all authors have read the manuscript in its present form.
